# Acute Endoplasmic Reticulum Stress-Independent Unconventional Splicing of XBP1 mRNA in the Nucleus of Mammalian Cells

**DOI:** 10.3390/ijms160613302

**Published:** 2015-06-10

**Authors:** Yuanyuan Wang, Pan Xing, Wenjing Cui, Wenwen Wang, Yanfen Cui, Guoguang Ying, Xin Wang, Binghui Li

**Affiliations:** 1 Laboratory of Cancer Cell Biology, National Clinical Research Center for Cancer, Tianjin Key Laboratory of Cancer Prevention and Therapy, Tianjin Medical University Cancer Institute and Hospital, Tianjin 300060, China; E-Mails: cristinaup@163.com (Y.W.); xingpan1314@126.com (P.X.); zuiaisuxueer@126.com (W.C.); wangwenwen.1988@163.com (W.W.); cuiyanfen@163.com (Y.C.); yingguoguang@163.com (G.Y.); 2 The First Department of Breast Tumor, National Clinical Research Center for Cancer, Tianjin Key Laboratory of Cancer Prevention and Therapy, Tianjin Medical University Cancer Institute and Hospital, Tianjin 300060, China

**Keywords:** ER stress, XBP1, unconventional splicing, unfolded protein response, IRE1α

## Abstract

The regulation of expression of X-box-binding protein-1 (XBP1), a transcriptional factor, involves an unconventional mRNA splicing that removes the 26 nucleotides intron. In contrast to the conventional splicing that exclusively takes place in the nucleus, determining the location of unconventional splicing still remains controversial. This study was designed to examine whether the unconventional spicing of XBP1 mRNA could occur in the nucleus and its possible biological relevance. We use RT-PCR reverse transcription system and the expand high fidelity PCR system to detect spliced XBP1 mRNA, and fraction cells to determine the location of the unconventional splicing of XBP1 mRNA. We employ reporter constructs to show the presence of unconventional splicing machinery in mammal cells independently of acute endoplasmic reticulum (ER) stress. Our results reveal the presence of basal unconventional splicing of XBP1 mRNA in the nucleus that also requires inositol-requiring transmembrane kinase and endonuclease 1α (IRE1α) and can occur independently of acute ER stress. Furthermore, we confirm that acute ER stress induces the splicing of XBP1 mRNA predominantly occurring in the cytoplasm, but it also promotes the splicing in the nucleus. The deletion of 5′-nucleotides in XBP1 mRNA significantly increases its basal unconventional splicing, suggesting that the secondary structure of XBP1 mRNA may determine the location of unconventional splicing. These results suggest that the unconventional splicing of XBP1 mRNA can take place in the nucleus and/or cytoplasm, which possibly depends on the elaborate regulation. The acute ER stress-independent unconventional splicing in the nucleus is most likely required for the maintaining of day-to-day folding protein homeostasis.

## 1. Introduction

In higher eukaryotes, the unfolded protein response (UPR)-associated adaptive system includes three different signaling pathways mediated by the endoplasmic reticulum (ER) stress sensors, inositol-requiring transmembrane kinase and endonuclease 1α (IRE1α), protein kinase-like ER kinase (PERK), and activation of transcription factor 6 (ATF6) [1,2]. Upon ER stress, IRE1α is activated and then initiates the unconventional splicing of the mRNA encoding X-box-binding protein-1 (XBP1) [3]. Only after the 26 nt intron within the open reading frame of the XBP1 mRNA is cleaved by IRE1α, the relegated XBP1 mRNA fragments form the XBP1s (spliced) mRNA that expresses a functional transcriptional factor, XBP1s [3,4].

In contrast to the conventional splicing that usually is catalyzed by the spliceosome and involves a consensus sequence at the flanks of introns according to the Chambon’s rule [5], the unconventional splicing of XBP1 mRNA is performed by IRE1α and an unknown RNA ligase [6,7]. The 26 nt intron of XBP1 mRNA also lacks the Chambon’s consensus sequence at its ends, and locates within a pair of characteristic stem-loop sequences. The conventional splicing exclusively occurs in the nucleus. However, it remains controversial where the unconventional splicing of XBP1 mRNA takes place. It is implied that the unconventional splicing of yeast HAC1 mRNA (yeast XBP1 homolog) could occur in both the nucleus and cytoplasm, although the acute ER stress-induced unconventional splicing of HAC1 mRNA is predominantly performed in the cytoplasm [8,9]. Because it is observed that IRE1α is localized in the inner nuclear membrane or translocated to the nucleus upon ER stress induction [10,11], the unconventional splicing of XBP1 mRNA in mammalian cells is also thought to take place in the nucleus. In contrast, there are many reports to directly show the occurrence of unconventional splicing of XBP1 mRNA in the cytoplasm [12,13,14].

XBP1 is required for the normal development of mice and is essential for plasma cell differentiation [15,16]. Increased XBP1s is implicated in human carcinogenesis, such as hepacellular carcinomas, multiple myelomas and breast tumors, and has been demonstrated to promote tumorigenesis [10,17,18]. Unfortunately, the weakly activated XBP1s is hard to detect in mammal cells. Here, we employ a XBP1 mRNA splicing-based ER stress sensor, ERAI (ER stress activated indicator) [19] to monitor ER stress in live cells, and unexpectedly, we observe an obvious basal splicing of ERAI mRNA without acute ER stress induction. Following this clue, we deploy experimental investigation, and our results and observations finally point to the basal unconventional splicing of XBP1 mRNA in the nucleus independent of acute ER stress induction. Following this clue, we deploy experimental investigation, and our results and observations finally reveal that the basal unconventional splicing of XBP1 mRNA in the nucleus can take place independently of and also be promoted by acute ER stress, and its occurrence appears to depend on the regulation of XBP1 mRNA structure involving its 5′-nucleotides sequence.

## 2. Results

### 2.1. The Presence of Acute ER Stress-Independent Unconventional Splicing of X-Box-Binding Protein-1 (XBP1) mRNA Sensor Sequence

**Figure 1:**
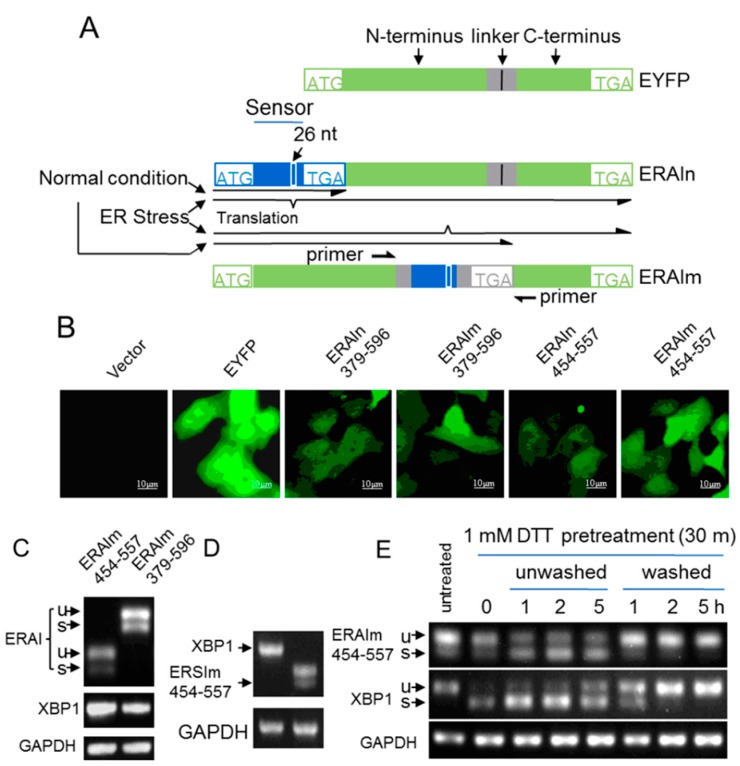
Acute ER (endoplasmic reticulum) stress-independent splicing of XBP1 mRNA sensor sequence. (**A**) Schematic representation of ER stress activation indicators (ERAI). Two polypeptide linkers of RSIAT and RPACKIPNDGKQKVMNH were inserted between the 154A–155D residues of EYFP proteins, and they did not affect the fluorescence of EYFP. ER stress sensor sequences (as indicated by the numbers in (**B**)) of XBP1 mRNA were cloned into the 5′-end or between the linkers of EYFP (ERAIn and ERAIm). These ER stress sensor sequences of XBP1 mRNA contain the 26 nt intron, and only after the intron is removed, the shifted reading frames will express fluorescent proteins; (**B**) The fluorescence of MCF-7 cells expressing vector, EYFP, ERAIn379–596, ERAIm379–596, ERAIn454–557 and ERAIm454–557; (**C**) The total RNA of MCF-7/ERAIm454–557 and MCF-7/ERAI379–596 cells without treatment were subjected to RT-PCR with XBP1 primers and ERAI primers; (**D**) The total RNA of MCF-7/ERAIm454–557 cells was subjected to RT-PCT with XBP1 or ERAI primers and the PCR products were run in the agarose gel under the same condition. The GAPDH was the loading control; and (**E**) MCF-7/ERAIm454–557 cells were pretreated with 1 mM DTT (dl-Dithiothreitol) for 30 min (time 0), and then DTT was removed (washed) or continued to exist (unwashed). The total RNA was subjected to RT-PCR. u, unspliced; s, spliced.

**Figure 2:**
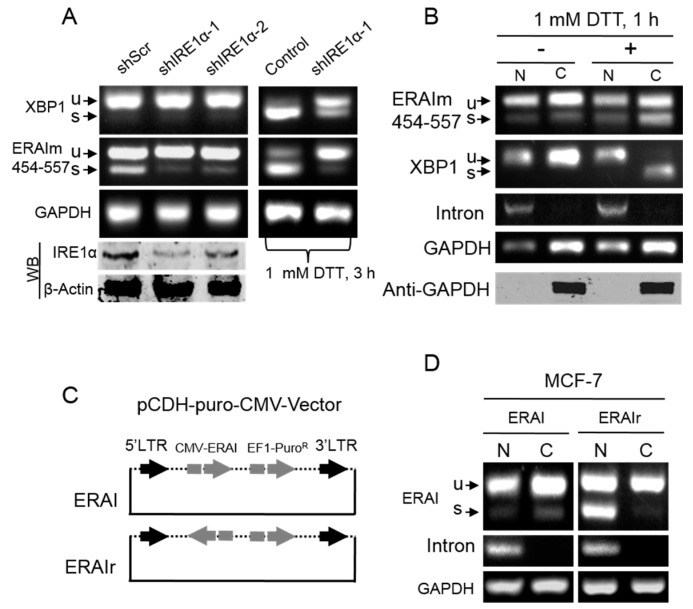
Acute ER stress-independent splicing of XBP1 mRNA sensor sequence in the nucleus. (**A**) IRE1α knockdown in MCF-7/ERAIm454–557 cells by two shRNA sequences. The bottom Western blot (WB) shows the knockdown effect. shScr is the scramble sequence as the control. The cells were treated with 1 mM DTT (**Right**) or vehicle (**Left**) for 3 h, and then the total RNA was subjected to RT-PCR with XBP1 primers and ERAI (ER stress activated indicator) primers; (**B**) MCF-7/ERAIm454–557 cells were fractioned after treatment with 1 mM DTT or vehicle for 1 h. The bottom WB shows the validation of fractions. Fifty micrograms of protein was loaded for each well; (**C**) Lentiviral constructs containing forward or backward CMV-ERAIm454–557, ERAI or ERAIr, (reverse ERAI) were stably expressed in MCF-7 cells; (**D**) Cells were cultured in the normal condition for 48 h, and then were fractioned. The fractioned RNA was subjected to RT-PCR with ERAI primers. GAPDH is a cytoplasmic protein; RT-PCR of intron indicates the pre-mRNA of XBP1 exclusively in the nucleus. N, nucleus; C, cytoplasm.

To conveniently investigate the mechanism underlying the unconventional splicing of XBP1 mRNA in mammalian cells, we introduced an ER stress indicator system (ER stress activated indicator, ERAI) [19] to our study. ERAI contained the ER stress sensor sequences of XBP1 mRNA, such as 379–596 and 454–557 nt (Figure S1A) [13,19], followed by EYFP (Enhanced Yellow Fluorescent Protein, EYFP), so we termed these indicators ERAIn (normal ER stress activated indicator) 379–596 and ERAIn454–557 (Figure 1A). In the presence of ER stress, ERAI mRNA was spliced and expressed a N-tagged EYFP fusion protein, while unspliced ERAI mRNA translated a protein without functional EYFP. Surprisingly, when stably expressed in MCF-7 cells, ERAIn379–596 and ERAIn454–557 showed significantly detectable fluorescence without acute ER stress induction (Figure 1B). To rule out the nonspecific translation that might contain intact EYFP, we inserted the 379–596 and 454–557 nt sequences of XBP1 mRNA into the middle of EYFP mRNA at the position corresponding to EYFP residues A154–D155, named ERAIm379–596 and ERAIm454–557 (Figure 1A). Only after ERAIm (mutant ER stress activated indicator) mRNA was spliced, it expressed a fluorescent protein containing the N- and C-fragments of EYFP (Figure 1A), because EYFP tolerated an insertion with linker flanks of RSIAT and RPACKIPNDGKQKVMNH (the sequence of amino acid) between residues A154 and D155 [20,21]. As shown in Figure 1B, all these indicator constructs displayed potent basal fluorescence without acute ER stress induction. It clearly demonstrated the occurrence of unconventional splicing of ERAIm mRNA. This was further confirmed by the RT-PCR results that significantly spliced mRNA of ERAIm379–596 or ERAIm454–557 was detected (Figure 1C). In contrast to spliced ERAIm mRNA, the spliced mRNA of endogenous XBP1 was almost undetectable under the same condition despite their comparable total mRNA levels (Figure 2D). Verified by sequencing (Figure S2), the 26 nt intron in the ERAIm454–557 mRNA was removed, indicating that the ER stress sensor sequence in ERAIm454–557 mRNA shared the same unconventional splicing with endogenous XBP1 mRNA. This provided us an ideal tool to study the basal unconventional splicing of sensor sequence in XBP1 mRNA without acute ER stress induction.

To explore whether the basal spliced ERAIm454–557 mRNA resulted from its high sensitivity to ER stress, which might exist in some cells at some time, we next compared the sensitivities of ERAIm454–557 and endogenous XBP1 mRNA to acute ER stress. We treated MCF-7/ERAIm454–557 cells with 1 mM DTT (DL-Dithiothreitol) for 30 min, and then assayed the splicing of ERAIm454–557 and endogenous XBP1 mRNA in the absence or continued presence of DTT at intervals. The results showed that the splicing of endogenous XBP1 mRNA was much more sensitive to acute ER stress than ERAIm454–557 mRNA (Figure 1E). After being pretreated with 1 mM DTT for 30 min (time 0 in Figure 1E), most of the endogenous XBP1 mRNA was spliced while ERAIm454–557 mRNA was nearly unaffected compared with the untreated control. During the continued treatment for 5 h, significantly spliced mRNA of ERAIm454–557 was also observed but was still less than that of endogenous XBP1. After the removal of treatment, the spliced XBP1 mRNA rapidly degraded concomitantly with the refreshment of unspliced XBP1 mRNA. At the same time, almost all the spliced ERAIm454–557 mRNA degraded during the restoring stage after acute ER stress (Figure 1E). These results showed that compared with endogenous XBP1 mRNA, ERAIm454–557 mRNA was less sensitive to acute ER stress-induced unconventional splicing but its already spliced form rapidly degraded during post-ER stress. These results ruled out the possibility that the basal splicing of ERAIm454–557 mRNA resulted from the finished or ongoing acute ER stress.

Furthermore, based on ERAI technology (Figure 1A), we generated a construct only containing the sequence of stem-loop pair including the 26 nt intron, termed ERAIm485–530 (Figure S1A). We stably expressed this gene in MCF-7 cells and measured its splicing in the absence of acute ER stress induction. As shown in Figure S1B, ERAIm485–530 still had a weak splicing in the absence of acute ER stress. This was confirmed by the fluorescent imaging (Figure S1C), suggesting the presence of spliced ERAIm485–530. Interestingly, this sequence was not responsive to acute ER stress induction, consistent with a previous report [13], although the endogenous XBP1 mRNA was (Figure S1B). Taken all together, our results revealed the presence of the machinery for unconventional splicing of XBP-1 mRNA sensor sequence independent of acute ER stress.

### 2.2. Basal Unconventional Splicing of XBP1 mRNA Sensor Sequence in ERAI Occurs in the Nucleus and Requires IRE1α

It was well-characterized that acute ER stress-dependent splicing of XBP1 mRNA was mediated by IRE1α [3]. Here, we tested whether the basal splicing of ERAIm454–557 mRNA also required IRE1α using shRNA constructs to knockdown IRE1α (Figure 2A bottom). As shown in Figure 2A (left), the knockdown of IRE1α significantly reduced the basal spliced mRNA of ERAIm454–557, suggesting the dependence of the basal splicing of ERAIm454–557 mRNA on IRE1α. In the validation controls, the knockdown of IRE1α also obviously suppressed acute ER stress-induced splicing of both ERAIm454–557 and endogenous XBP1 mRNA (Figure 2A right).

Acute ER stress-dependent unconventional splicing of XBP1 mRNA was proved to predominantly occur in the cytoplasm [14]. Here, we separated nuclear and cytoplasmic fractions of MCF-7/ERAIm454–557 cells and extracted their total RNA, respectively. The RT-PCR results showed that the spliced mRNA of ERAIm454–557 was found in both the nucleus and cytoplasm in the absence of acute ER stress induction (Figure 2B). The relative level of spliced ERAIm454–557 mRNA in the nuclear fraction was comparable to that in the cytoplasmic fraction, and so the cytoplasmic spliced ERAIm454–557 mRNA most likely was exported from the nucleus like unspliced XBP1 and GAPDH mRNA. In the acute ER stress condition, the spliced mRNA of both ERAIm454–557 and endogenous XBP1 was significantly increased in the cytoplasm but not in the nucleus. These results not only confirmed that acute ER stress-induced XBP1 mRNA splicing predominantly took place in the cytoplasm but also verified our separation of the nuclear and cytoplasmic fractions. The separation was further confirmed by the Western blot results that the nuclear fraction did not contain detectable cytoplamic GAPDH protein and the intron RT-PCR results that indicated the pre-mRNA of XBP1 exclusively located in nuclear fraction (Figure 2B). These results strongly suggested that the basal splicing of ERAIm454-557 mRNA occurred in the nucleus.

To further confirm the occurrence of unconventional splicing of ERAIm454-557 mRNA in the nucleus, we generated a lentiviral construct containing backward CMV-ERAI454-557, termed ERAIr (reverse ERAI) (Figure 2C). ERAIr keeps all the elements between 5′-LTR and 3′-LTR, and thus allows establishing stable cell lines. However, cells carrying ERAIr transcribe CMV-driven forward ERAI mRNA without termination signal that cannot be efficiently exported from nucleus [22], which leads to the accumulation of ERAI mRNA in the nucleus. This construct offers a chance to directly observe the splicing of mRNA in the nucleus without cytoplasmic interfering. As expected, MCF-7/ERAIr cells showed obvious ERAI mRNA splicing in the nucleus not in the cytoplasm (Figure 2D), and also no fluorescence was observed (data not shown). It was of note that an unspliced ERAI mRNA band was also detected in the cytoplasm of MCF-7/ERAIr cells. This should result from the reverse complement sequence of ERAI contained in the 5′-LTR–3′-LTR RNA whose transcription is initiated at 5′-LTR and terminated at 3′-LTR [23]. The properly processed 5′-LTR–3′-LTR RNA is exported to the cytoplasm, the reverse complement sequence of ERAI mRNA of which cannot be unconventionally spliced but be detected using RT-PCR. Taken together, these results clearly demonstrated that the basal unconventional splicing of XBP1 mRNA sensor sequence in ERAI occurred in the nucleus independent of acute ER stress, suggesting the existence of the basal unconventional machinery of XBP1 mRNA in the nucleus.

### 2.3. The 5′ End Nucleotide Sequence Is Critical to the Unconventional Splicing of XBP1 mRNA

**Figure 3:**
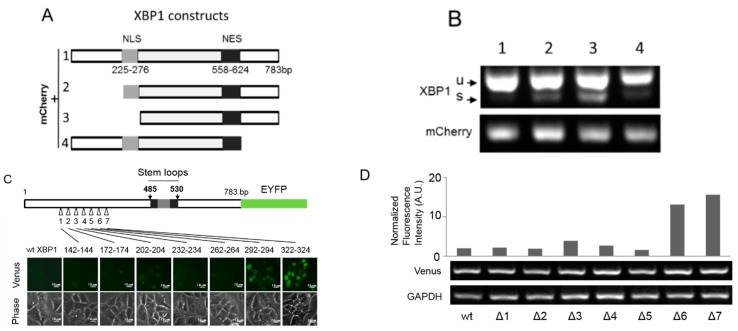
Effects of flank sequences of stem-loop pair on the splicing of XBP1 mRNA. (**A**) Constructs of mCherry followed by XBP1 fragments. The number indicates the position of nucleotides in unspliced XBP1. NLS, putative nuclear localization sequence; NES, putative nuclear export sequence; (**B**) The total RNA of MCF-7 cells expressing the constructs in (**A**) was subjected to RT-PCT with XBP1 primers and mCherry primers; (**C**) Effect of 5′-nucleotide sequence of XBP1 mRNA on its basal splicing (scale bar = 10µm). XBP1-EYFP was constructed as ERAI1-783, as described in Figure 1A. ERAI1-783 cDNAs with Different deletions in the 5′-end sequence of XBP1 were respectively stably expressed in MCF-7 cells. Δ1, 2, 3, 4, 5, 6, and 7 represent seven deletion mutants. Fluorescence images indicate the basal unconventional splicing; and (**D**) The normalized fluorescence intensity in (**C**) was presented as the ratio of fluorescence intensity/ERAI1-783 expression level. Venus RT-PCR indicates the expression level of ERAI1-783 constructs. The value is obtained from the mean of one representative triplicate experiment.

The basal unconventional splicing of ERAIm454–557 mRNA was much more significant than that of endogenous XBP1 mRNA (Figure 1C–E), implying the importance of the sensor sequence containing the 26 nt intron in XBP1 mRNA in the unconventional splicing. To obtain more information about the basal splicing of XBP1 mRNA, we generated different constructs containing truncated XBP1 fragments fused with mCherry at *N*-terminus (Figure 3A). We stably expressed these mCherry-tagged XBP1 fragments in MCF-7 cells (Figure S3), and found that the truncations of 5′-nucleotides significantly increased the splicing of XBP1 mRNA in the absence of acute ER stress (Figure 3B). Therefore, we further explored the effect of 5′ end sequence of XBP1 mRNA on its unconventional splicing. We generated a series of deletion mutants in 5′ end of XBP1 cDNA followed by EYFP cDNA, which expressed an EYFP fusion protein upon XBP1 mRNA unconventional splicing according to ERAI technology (Figure 1A). To avoid the frame shift, we omitted three base pairs in each mutant (Figure 3C). The first five deletions in the 5′ end sequence of XBP1 did not affect their basal unconventional splicing, whereas the deletion of 292–294 or 322–324 bp in XBP1 mRNA significantly increased its basal splicing (Figure 3C,D). These results suggest that the 5′ nucleotide sequence of XBP1 mRNA is critical to its unconventional splicing and most likely forms some special secondary structure that might act as a platform for some RNA-binding protein(s). The endogenous XBP1 mRNA in MCF-7 cells possibly blocked its sensitivity to the basal unconventional splicing machinery in the nucleus by forming some secondary structure involving its 5′-nucleotides.

### 2.4. The Basal Unconventional Splicing of Endogenous XBP1 mRNA Occurs in the Nucleus Independent of ER Stress

**Figure 4:**
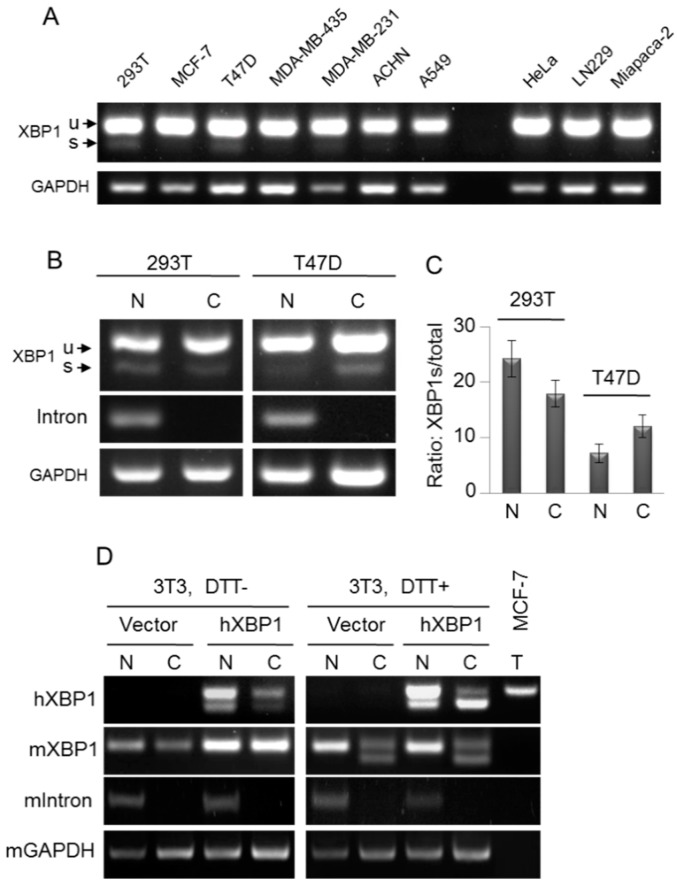
Occurrence of the unconventional splicing of endogenous XBP1 mRNA in the nucleus. (**A**) Cells were cultured in the normal condition. The total RNA was subjected to RT-PCR with XBP1 primers; (**B**) 293T and T47D cells were cultured in the normal condition, and then were fractioned. The fractioned RNA was subjected to RT-PCR with XBP1 primers; (**C**) Quantified fractions of nuclear spliced XBP1 mRNA in 293T and T47D cells, the ratio of spliced XBP1 mRNA to total XBP1 mRNA. Data are from three independent experiments as described in (**B**). Error bars show means ± SD; and (**D**) Human XBP1 (hXBP1) was stably expressed in mouse 3T3 cells. Cells were treated with 1 mM DTT or vehicle for 1 h, and then were fractioned. The fractioned RNA was subjected to RT-PCR with XBP1 primers. The primers are specific respectively for hXBP1 and mXBP1 RT-PCT. mIntron RT-PCR indicates the pre-mRNA of mXBP1 exclusively in the nuceus. N, nucleus; C, cytoplasm; T, MCF-7/wt total RNA used as the control for RT-PCR of hXBP1 mRNA.

Now, we are wondering whether the basal unconventional splicing of endogenous XBP1 mRNA in the nucleus occurs in mammalian cells. To obtain model cell lines with relatively high levels of basally spliced endogenous XBP1 mRNA, we tested the basal spliced XBP1 mRNA in a variety of cell lines, and found that 293T and T47D cells exhibited a detectable background spliced XBP1 mRNA in the normal cell culture condition (Figure 4A). After separation, significantly spliced XBP1 mRNA was detected in the nuclear fractions of 293T and T47D cells (Figure 4B,C), suggesting the occurrence of the basal unconventional splicing of endogenous XBP1 mRNA in the nucleus in the absence of acute ER stress. However, the spliced XBP1 level is very low, because a high level of spliced XBP1 is toxic to cells.

To further confirm that the basal unconventional splicing of the intact XBP1 mRNA can occur in the nucleus under some conditions, we stably expressed human XBP1 (hXBP1) in mouse 3T3 cells, and RT-PCR can easily distinguish human and mouse XBP1 (mXBP1) mRNAs. Given that some RNA-binding protein(s) is required to block the basal unconventional splicing of endogenous XBP1 mRNA as implied by the results in Figure 3, hXBP1 mRNA in mouse 3T3 cells could be more susceptible to the unconventional splicing machinery in the nucleus, because the heterogeneous interaction between human XBP1 mRNA and mouse RNA-binding protein(s) is probably decreased. As shown in Figure 4D, 3T3/hXBP1 cells showed no detectable spliced endogenous mXBP1 mRNA in both the nucleus and cytoplasm, whereas the spliced exogenous hXBP1 mRNA was apparently observed in the nucleus. In the condition of acute ER stress, the spliced endogenous mXBP1 RNA dramatically increased in the cytoplasm but not in the nucleus. Similarly, acute ER stress significantly promoted the splicing of exogenous hXBP1 in the cytoplasm while almost leaving the nuclear splicing unaffected. These results clearly showed that the unconventional splicing of the intact human XBP1 mRNA can potentially take place in the nucleus independent of acute ER stress under some conditions.

### 2.5. ER Stress Promotes the Unconventional Splicing of XBP1 mRNA in the Nucleus

**Figure 5:**
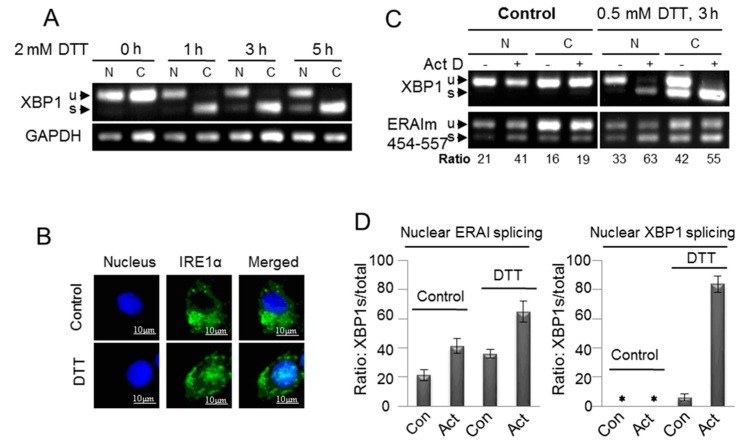
Promotion of the unconventional splicing of XBP1 mRNA in the nucleus by acute ER stress. (**A**) MCF-7/wt cells were fractioned after they were treated with 2 mM DTT for different time as indicated. The fractioned RNA was subjected to RT-PCR with XBP1 primers; (**B**) MCF-7/wt cells were treated with 2 mM DTT for 5 h, and then were fixed. The immunoflurorescence of the fixed cells stained with monoclonal IRE1α antibody was imaged, as described in the Methods; (**C**) MCF-7/ERAIm454–557 cells were fractioned after they were treated with 0.5 mM DTT and/or 10 μg/mLK Actinomycin D (Act D) (pretreatment for 1 h) or vehicle for 3 h. The ratio shows the percentage of spliced mRNA in total ERAIm454–557 mRNA including spliced and unspliced mRNA; and (**D**) Quantified fractions of nuclear spliced XBP1 or ERAI mRNA, the ratio of spliced XBP1 mRNA to total XBP1 mRNA. Data are from three independent experiments as described in (**C**). Error bars show means ± SD. * means there was no stripe.

To further investigate whether the unconventional splicing of endogenous XBP1 mRNA in the nucleus was associated with acute ER stress, we treated MCF-7/wt cells with a high concentration of DTT (2 mM) for the time course analysis. Upon the induction of acute ER stress, the spliced XBP1 mRNA dramatically increased to the saturation level in the cytoplasm while gradually rising over time in the nucleus (Figure 5A). This suggested that acute ER stress promoted the unconventional splicing of XBP1 mRNA in the nucleus. Since our results showed that the unconventional splicing machinery in both nucleus and cytoplasm required IRE1α (Figure 2A), we tested whether acute ER stress increased the localization of IRE1α to the nucleus. The immunofluorescent imaging results showed that DTT treatment significantly induced the nuclear translocation of IRE1α (Figure 5B), which was consistent with the previous report [11].

### 2.6. The De Novo Transcription Is Not Required for the Unconventional Splicing of XBP1 mRNA in the Nucleus

To determine the requirement of *de novo* transcription for the unconventional splicing in the nucleus, we used actinomycin D (Act D) to block transcription in MCF-7/ERAIm454-557 cells. At a high concentration, Act D intercalates into DNA and inhibits all three classes of RNA polymerase transcription [24,25]. Since we loaded the same amount of total RNA for RT-PCR analyses, the ratio, not the level, of spliced mRNA provided useful information after the transcription was blocked. Our results showed that Act D did not repress, but actually increased, the ratio of spliced ERAI and XBP1 mRNA in both the nucleus and cytoplasm under the condition of ER stress (Figure 5C,D). Therefore, like the unconventional splicing of XBP1 mRNA in the cytoplasm [14], the nuclear unconventional splicing also did not require *de novo* transcription.

Besides, the results obtained with *de novo* transcription blockage afforded by Act D (Figure 5C,D) make several important points. *De novo* transcription blockage abolished the supplement of unspliced XBP1 mRNA, and thus increased the ratio of unconventionally spliced mRNA, because the existed mRNA was continuously spliced given the presence of the unconventional splicing machinery. We observed that *de novo* transcription blockage increased the ratio of nuclear spliced ERAI mRNA in the absence of acute ER stress (Figure 5C,D), and it confirmed the presence of the basal unconventional splicing machinery in the nucleus (Figure 1 and Figure 2). Acute ER stress enhanced the spliced ERAI mRNA in the nucleus (Figure 5C,D), and this possibly resulted from the acute ER stress-induced nuclear translocation of IRE1α (Figure 5B). De novo transcription blockage exerted the similar stimulative effect (two-fold increase) on the nuclear spliced ERAI mRNA regardless of acute ER stress (Figure 5C,D), and it suggested that acute ER stress did not increase the sensitivity of ERAI mRNA to the nuclear unconventional splicing machinery. *De novo* transcription blockage did not increase the ratio of nuclear spliced XBP1 mRNA without acute ER stress induction (Figure 5C,D), and it implied the insensitivity of endogenous XBP1 mRNA to the basal nuclear unconventional splicing machinery, which was also supported by our results in Figure 1C,D and Figure 2A,B). Acute ER stress increased the nuclear spliced endogenous XBP1 mRNA (Figure 5C,D), and thus it potentially facilitated the nuclear unconventional splicing of XBP1 mRNA, consistent with the speculation from Figure 5A. Interestingly, *de novo* transcription blockage dramatically increased the ratio of nuclear spliced XBP1 mRNA in the presence of acute ER stress (Figure 5C,D), and this showed that in the condition of acute ER stress, endogenous XBP1 mRNA was sensitive to the nuclear unconventional splicing machinery. In fact, the similar result was also observed in the cytoplasm (Figure 5C). This was consistent with the result in Figure 1E. However, why did we fail to observe the significant fractions of nuclear spliced endogenous XBP1 mRNA in the absence of de novo transcription blockage (Figure 5A,C)? One possibility was that the supplement of unspliced mRNA from *de novo* transcription was very effective so that it largely decreased the ratio of nuclear spliced endogenous XBP1 mRNA. This speculation was supported by our results in Figure 1E.

### 2.7. XBP1s Promotes the Growth of MCF-7 Cells

**Figure 6:**
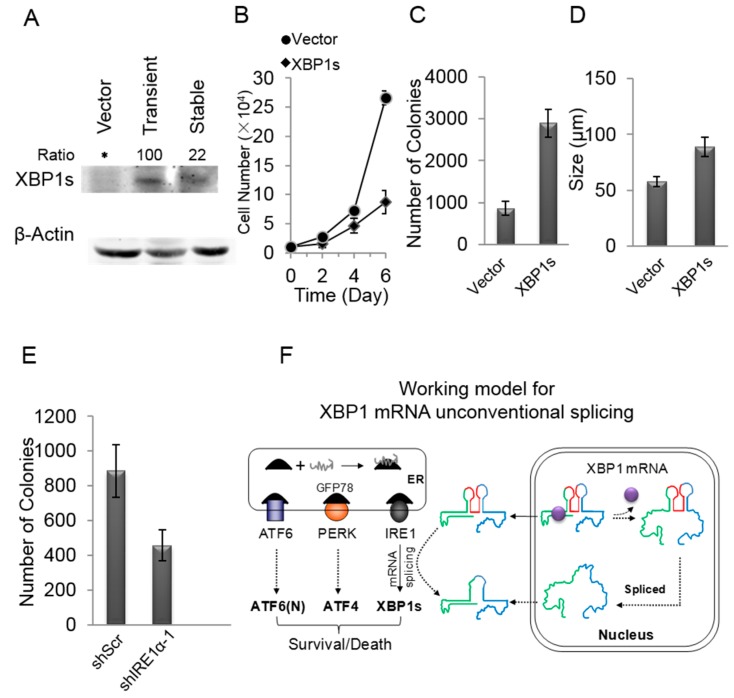
A low level of XBP1s promoted the growth of MCF-7 cells. (**A**) MCF-7 cells were infected lentivirus expressing XBP1s or empty vector. Cells in Passage 1 (transient) expressed a high level of XBP1s, while the adapted cells (stable) after Passage 1 expressed a low level of XBP1s, * means there was no stripe; (**B**) The growth rate of MCF-7/Vector and adapted MCF-7/XBP1s cells. Data are from three independent triplicate experiments. Error bars show means ± SD; (**C**,**D**) The soft agar colonies formation of MCF-7/Vecotr and adapted MCF-7/XBP1s cells. The colony numbers (**C**) and sizes (**D**) are shown. Data are from three independent triplicate experiments. Error bars show means ± SD; (**E**) The colony number of MCF-7/shScr and MCF-7/shIRE1α-1 cells. shScr is the scramble sequence. shIRE1α-1 is the knockdown sequences of IRE1α, as verified in Figure 2A. Data are from three independent triplicate experiments. Error bars show means ± SD; and (**F**) Working model for XBP1 mRNA unconventional splicing. XBP1 mRNA is exported into the cytoplasm and undergoes acute ER stress-dependent splicing, but XBP1 mRNA also can be subjected to ER stress-independent splicing in the nucleus depends on its structure. Green and blue lines, respectively, present the 5′- and 3′-nucleotides of XBP1 mRNA. Red line represents the 26 nt intron. Purple ball represents RNA-binding protein that could regulate the structure of XBP1 mRNA for the nuclear unconventional splicing.

XBP1 usually existed as an unspliced form, XBP1u. XBP1s was reported to promote tumorigenesis [17,26], and here we further tested the effect of XBP1s on MCF-7 cells, a non-invasive breast cancer cell line, where XBP1s could not be detected (Figure 6A). MCF-7 cells were infected with lentivirus expressing XBP1s or empty vector for 48 h, and then these cells were transplanted and amplified in dishes. We found that the cells expressing XBP1s took two days to attach to the culture dish in the first passage, whereas the control cells attached normally within hours (data not shown). However, MCF-7/XBP1s cells adapted soon and attached normally after the second passage. The Western blot results showed that the level of XBP1s in the adapted MCF-7/XBP1s cells was significantly lower than that in the transiently infected cells (Figure 6A). This suggested that a high level of XBP1s was disadvantageous to MCF-7 cells, but MCF-7 cells adapted soon by decreasing the expression of XBP1s. However, the adapted MCF-7/XBP1s grew much faster than the control cells (Figure 6B). The anchorage-independent growth in soft agar was also boosted in adapted MCF-7/XBP1s cells compared with the control cells, and both number and size of colonies were significantly enhanced in MCF-7/XBP1s cells (Figure 6C,D). In contrast, shIRE1α significantly suppressed the colonies formation ability of MCF-7 cells (Figure 6E), indicating the importance of XBP1s in MCF-7 cells. Furthermore, we expressed XBP1s in a normal breast cell line, MCF-10A, and all the cells failed to attach to the culture dish and finally died after they were transplanted (data not shown). These data showed that the unregulated XBP1s was toxic to cells, as the heavy ER stress did [27]. The typical IRE1α-XBP1 pathway in the cytoplasm/ER is activated after ATF6 and PERK pathways [27], and thus cytoplasmic XBP1 mRNA splicing represents a severe response to acute ER stress.

## 3. Discussion

The unconventional splicing of XBP1 mRNA is well-characterized during ER stress [14,28]. However, it remains controversial whether the unconventional splicing takes place in the cytoplasm, nucleus or both. Therefore, investigating the location of unconventional splicing is very important to understand its biological relevance. At present, it has been demonstrated that the acute ER stress-induced unconventional splicing of XBP1 mRNA predominantly occurs in the cytoplasm and is mediated by the ER-located endonuclease IRE1α and an unknown ligase [6,14]. Nonetheless, there is still some evidence implying that the unconventional splicing of XBP1 mRNA could also take place in the nucleus. This speculation is supported by the observations that IRE1α is located to the inner nuclear envelope and that IRE1α is translocated to the nucleus to cleave mRNA substrates upon ER stress [10,11].

Since the very low level of XBP1s in mammal cells under the normal conditions is difficult to be detected, and it largely prevents the investigation on the location of XBP1 mRNA without acute ER stress induction. Herein, with the aid of ERAI and ERAIr reporters, using MCF-7 cells as the model, we clearly demonstrate that the basal unconventional splicing machinery exists in the nucleus and also requires IRE1α (Figure 1 and Figure 2). Since in the same condition we fail to detect the significant splicing of endogenous XBP1 mRNA in the nucleus of MCF-7 cells, we analyze the effects of the sequence of XBP1 mRNA on its unconventional splicing, and reveal that the 5′-nucleotide sequence of XBP1 mRNA blocks its sensitivity to the basal unconventional splicing machinery in the absence of acute ER stress (Figure 3). However, we still observe that the endogenous XBP1 mRNA can be potentially spliced in the nucleus without acute ER stress induction in some cell lines, such as 293T and T47D cells, or in some conditions (Figure 4). This suggests that the basal unconventional splicing of the endogenous XBP1 mRNA can also take place in the nucleus.

Interestingly, the unconventional splicing of XBP1 and ERAI mRNA in the nucleus is also obviously promoted by acute ER stress (Figure 5). In the meantime, we also observe that the nucleus-localized IRE1α significantly increased (Figure 5B), consistent with the previous report [11]. Therefore, the acute ER stress possibly promotes the occurrence of unconventional splicing of XBP1 mRNA in the nucleus by inducing translocation of IRE1α to the nucleus. Although this speculation is compatible with our results that the unconventional splicing in the nucleus also depends on IRE1α, it could not explain why the basal unconventional splicing machinery in the nucleus inefficiently splices the endogenous XBP1 mRNA in MCF-7 cells while it significantly works on ERAI mRNA in the absence of acute ER stress (Figure 1, Figure 2 and Figure 5). Considering that the endogenous XBP1 mRNA always carries its 5′-nucleotide sequence that represses its nuclear splicing sensitivity (Figure 3), acute ER stress most likely additionally relieves the blockage of the 5′-nucleotide sequence of XBP1 mRNA by regulating its secondary structure. This speculation is also supported by the results obtained with *de novo* transcription blockage (Figure 5C,D) that acute ER stress dramatically increased the nuclear spliced XBP1 mRNA when newly synthesized XBP1 mRNA was abolished, while the nucleus spliced ERAI mRNA was not increased apparently. This is because that ERAI mRNA does not contain the 5′-nucleotide sequence of XBP1 mRNA, so its nuclear splicing fails to respond to acute ER stress regulation. Our results show that the blockage of *de novo* transcription does not repress, but actually increases, the unconventional splicing of ERAI and XBP1 mRNA in the nucleus (Figure 5C), and thus rules out the possibility that some of the newly synthesized XBP1 mRNA somehow properly unfolding undergo basal nuclear unconventional splicing, which is supported by some evidence in yeasts [8]. Therefore, acute ER stress promotes the nuclear splicing of XBP1 mRNA possibly mainly by regulating the structure of XBP1 mRNA, and in particular of its 5′-nucleotide sequence.

Here, our results show that the forced expression of XBP1s is toxic to epithelial MCF-10A cells (data not shown), consistent with observations that the sustained or heavy ER stress induces cell death [27]. The high level of XBP1s is also disadvantageous to MCF-7 cells. In contrast, MCF-7 cells expressing a low level of XBP1s grow faster in both anchorage-dependent and -independent platforms, compared with the control cells (Figure 6B–E). This clearly implicates that the low level of XBP1s facilitates the growth of cancer cells. Indeed, cancer cells are frequently reported to have an elevated level of spliced XBP1 relative to normal cells [29,30], and moreover, the conditioned expression of XBP1s promotes tumorigenesis [17]. In fact, besides cancer, a variety of human diseases including metabolic disease, inflammatory disease and neurodegenerative disease are associated with UPR, and they usually progress over years and thus are thought to adapt to chronic ER stress [29,31,32]. Nonetheless, where the day-to-day spliced XBP1s comes from and how cells control the spliced XBP1s to a low level remains elusive. In the current study, our results suggest that the basal unconventional splicing of XBP1 mRNA can occur in the nucleus independently of acute ER stress that induces unconventional XBP1 mRNA splicing predominantly taking place in the cytoplasm, which seems to rely on the secondary structure of XBP1 mRNA. In addition, when ERAIm454–557 is expressed in several cell lines including cancer cell lines, such as MCF-7, MDA-MB-231, HeLa, Du145, PC3 and HCT116, and normal cell lines, such as NIH3T3, HLB-100 and MCF-10A, obvious fluorescence is observed in the normal cell culture condition (data not shown), and therefore, the basal machinery underlying acute ER stress-independent splicing of XBP1 mRNA possibly represents an ubiquitous phenomenon. Some cells possibly modulate the structure of XBP1 mRNA in the nucleus to maintain an increased basal level of XBP1s without acute ER stress, for example, via some RNA-binding protein(s). The basal splicing of XBP1 mRNA in the nucleus may reflect the so-called “chronic ER stress”.

The activation of typical IRE1α-XBP1 pathway in the cytoplasm/ER is posterior to that of ATF6 and PERK pathways [27], and thus cytoplasmic XBP1 mRNA splicing represents a full response to acute ER stress, and it can finally induce cell death by regulating a subset of genes expression [33,34]. In fact, the functional ATF6 and PERK pathways, not the IRE1α-XBP1 branch, are critical to coping with acute ER stress for cell survival [35]. However, unlike ATF6 and PERK, which are not essential genes, IRE1α (or its XBP1 effector) is indispensable to embryonic development [36,37]. Growing cells often have active protein synthesis, and thus often suffer from unfolded proteins to some extent. If they are always initiating acute ER stress response, the increased death risk should be a big challenge. Therefore, the acute ER stress-independent unconventional splicing (activation) of XBP1 mRNA in the nucleus may reflect a sort of mild UPR and account for the day-to-day protein folding homeostasis. This could be the physiological relevance of the basal unconventional splicing in the nucleus. It would be of interest to further figure out the mechanism underlying unconventional splicing of XBP1 mRNA in the nucleus, which may help us further understand the role of XBP1s in human diseases and the relevant clinical applications.

## 4. Materials and Methods

### 4.1. Plasmid Construction

ERAIn constructs contain parts of human XBP1 cDNA sequences followed by EYFP. ERAIm constructs have parts of human XBP1 cDNA inserted into EYFP at the position corresponding to EYFP residues 154A–155D with flank linkers of RSIAT and RPACKIPNDGKQKVMNH. The full cDNA of XBP1 (1157 nt) was cloned from mRNA in MCF-7 cells using a cDNA generation kit (Biophay, Beijing, China). The cDNA of XBP1s was generated from XBP1 cDNA by removing the 26 nt intron using PCR. All ERAI cDNAs were generated by PCR, and cloned into the lentiviral expressing vectors, pCDH-puro-CMV or pCDH-Neo-CMV using the eFusion recombinant cloning kit (Biophay, Beijing, China).

The pLKO.1 lentiviral RNAi expression system was used to construct lentiviral shRNA. The shRNA sequences used in this study included the following:

shScr (Scramble shRNA): CCTAAGGTTAAGTCGCCCTCG.
shIRE1α-1: GCACGTGAATTGATAGAGAAG.
shIRE1α-2: CCCATCAACCTCTCTTCTGTA.

### 4.2. RNA Isolation and RT-PCR

Cells were cultured and treated as indicated in the content and then collected. Total RNA was extracted with a TRIzol procedure as specified by the manufacturer (Invitrogen, Carlsbad, CA, USA). Purified RNA was quantified by spectrophotometry. The splicing of ERAI and XBP1 RNAs was detected by standard RT-PCR (reverse transcription system and the expand high fidelity PCR system (Thermo, Massachusetts, MA, USA)) using a random primer and then the specific primers as following:

GAPDH-F: GAAGGTGAAGGTCGGAGTC.
GAPDH-R: GAAGATGGTGATGGGATTTC.
Human XBP1-F: GAATGAAGTGAGGCCAGTGG.
Human XBP1-R: ACTGGGTCCTTCTGGGTAGA.
Mouse XBP1-F: ACGAGGTTCCAGAGGTGGAG.
Mouse XBP1-R: AAGAGGCAACAGTGTCAGAG.
ERAIm-F: ATCGACTTCAAGGAGGACGGCAACA (for all ERAIm constructs).
ERAIm454–557-R: TTCTGGAGGGGTGACAACTGG (for ERAIm375–596 and ERAIm454–557).
ERAIm485–530-R: GTTCATCACCTTCTGCTTGCCG (for ERAIm485–530, ERAIm485–596 and ERAIm373–530).
mCherry-F: ATGGTGAGCAAGGGCGAGG.
mCherry-R: GACAGGATGTCCCAGGCGAA.
GAPDH mRNA level was used as the normalization control.

### 4.3. Cellular Fraction

Cells were fractionated as described [14]. In brief, MCF-7 cells grown on 100-mm dishes were collected using a cell scraper, and incubated in 0.5% NP40-PBS solution on ice for 10 min to solubilize the plasma membrane. After centrifugation at 500× *g* for 5 min at 4 °C, the precipitate containing the nuclei was separated from the supernatant containing the cytoplasm, and then were washed with ice-cold 0.5% NP40-PBS three times. The cytoplasmic and nuclear fractions were subjected to total RNA extraction with TRIzol (Invitrogen, Carlsbad, CA, USA). The RT-PCR reactions of nuclear and cytoplasmic RNA were carried out as described in the section “RNA Isolation and RT-PCR”.

### 4.4. Cell Culture

Cancer Cells, HBL-100 and 3T3 cells were maintained in DMEM (high glucose) supplemented with 10% fetal bovine serum (Hyclone, Logan, UT, USA) and 50 IU penicillin/streptomycin (Invitrogen, Carlsbad, CA, USA), and MCF-10A cells were cultured in DMEM/F12 containing 5% horse serum, 20 ng/mL EGF, 0.5 mg/mL hydrocortisone, 100 ng/mL cholera toxin, 10 μg/mL insulin and 50 IU penicillin/streptomycin in a humidified atmosphere with 5% CO_2_ at 37 °C.

For cell growth assay, 10,000 cells were seeded in each well of 12-well plates, and were counted from the second day (Day 0, the day seed cells). The numbers of cells were normalized to compare the growth rate.

### 4.5. Anchorage-Independent Cell Culture

For soft agar assay, 10,000 cells suspended in top agarose solution (0.3%) were poured over bottomed agarose (0.6%) previously solidified in 6-well plates. Cells were cultured in a humidified atmosphere with 5% CO_2_ at 37 °C for weeks, and then colonies were counted and imaged for size calculation.

### 4.6. Imaging of Cells

For immunofluorescent imaging, cells growing on coverslips were fixed with 4% paraformaldehyde at room temperature for 15 min. The cells were then washed three times with PBS, permeabilized in 0.1% Triton X-100 at room temperature for 2 min, and washed three times with PBS. The fixed samples were blocked in PBS containing 5% milk for 30 min at room temperature and then incubated with anti-IRE1α monoclonal antibody (1/500 dilution) (Santa Cruz Biotechnology, Santa Cruz, CA, USA) for 1 h at room temperature. After being washed with PBS, the samples were reacted with FITC-conjugated goat anti-mouse IgG (1/500 dilution, Jackson ImmunoResearch Laboratories, Pennsyivania, PA, USA) for 1 h at room temperature. The samples were then treated with 2 μg/mL of Hoechst33258 for 5 min at room temperature. Finally, the coverslips were washed three times with PBS, placed on a glass slide, and sealed with transparent nail polish. The images were captured with a Nikon Eclipse TE2000-U fluorescence microscope (Nikon Corporation, Tokyo, Japan). For live cells imaging, cells were grown on 6-well plates, and after the desired treatments, the fluorescence and phase-contrast imaging was processed.

### 4.7. Western Blot

After desired treatments as specified in the Results section, cells were washed twice with PBS and lysed in buffer (20 mM Tris-HCl, pH 7.5, 150 mM NaCl, 1 mM EDTA, 1% Triton X-100, 2.5 mM sodium pyrophosphate, 1 mM b-glycer-ophosphate, 1 mM sodium vanadate, 1 mg/mL leupeptin, and 1 mM phenylmethyl-sulfonylfluoride). Equal amounts of protein were loaded. Western detection was carried out using a Li-Cor Odyssey image reader. The goat anti-mouse immunoglobulin G (IgG) and goat anti-rabbit IgG secondary antibodies were obtained from Li-Cor.

### 4.8. Lentivirus Production

Viral packaging was done according to the previously described protocol [38]. Briefly, expressing plasmids pCDH-CMV-cDNA, pCMV-dR8.91, and pCMV-VSV-G were co-transfected into 293T cells using the Calcium Phosphate method at 20:10:10 μg (for a 10-cm dish). The transfection medium containing calcium phosphate and plasmid mixture was replaced with fresh complete medium after incubation for 5 h. Media containing virus was collected 48 h after transfection and then concentrated using Virus Concentrator Kit (Biophay, Beijing, China). The virus finally dissolved in the proper amount of complete growth medium and stocked at −80 °C. Cancer cells were infected with the viruses at the titer of 100% infection in the presence of Polybrene (10 μg/mL) for 48 h, and then cells were selected with puromycin or neomycin.

## 5. Conclusions

Our results show that the unconventional splicing of XBP1 mRNA can occur in both the nucleus and cytoplasm of mammal cells (Figure 6F). The nuclear unconventional splicing of XBP1 mRNA can take place independently of, and also be promoted by, acute ER stress, and its occurrence appears to depend on the regulation of the XBP1 mRNA structure involving its 5′-nucleotides sequence.

## Supplementary Materials

Supplementary materials can be found at http://www.mdpi.com/1422-0067/16/06/13302/s1.

## Abbreviations

XBP1: X-box-binding protein-1
ER: endoplasmic reticulum
IRE1α: inositol-requiring transmembrane kinase and endonuclease 1α
UPR: unfolded protein response
PERK: protein kinase-like ER kinase
ATF6: activation of transcription factor 6
ERAI: ER stress activated indicator
RT-PCR: reverse transcription system and the expand high fidelity PCR system
