# Supplementary material for: Acute Endoplasmic Reticulum Stress-Independent Unconventional Splicing of XBP1 mRNA in the Nucleus of Mammalian Cells
Source: Int J Mol Sci. 2015 Jun 10;16(6):13302–21. doi: 10.3390/ijms160613302 (PMC4490496; doi:10.3390/ijms160613302)
Supplement: Supplementary file 1 [file ijms-16-13302-s001.pdf]

Supplementary Information

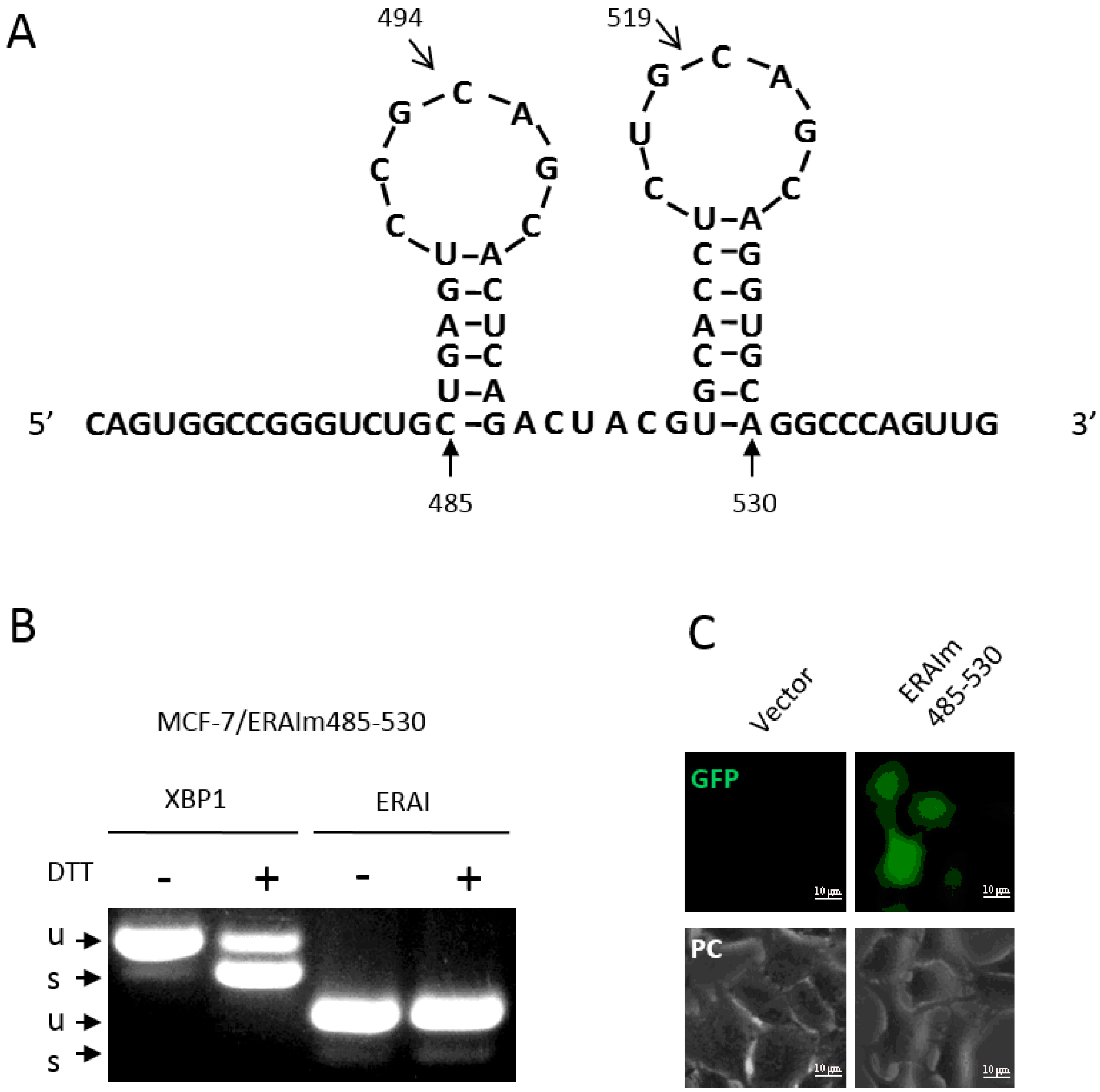

Figure S1. Expression of ERAIm485–530 in MCF-7. (**A**) The sequence of ERAIm485–530; (**B**) The total RNA of MCF-7/ERAIm485–530 cells was subjected to RT-PCT with XBP1 or ERAI primers and the PCR products were run in the agarose gel under the same condition; and (**C**) The fluorescence of MCF-7 cells expressing vector and ERAIm485–530.

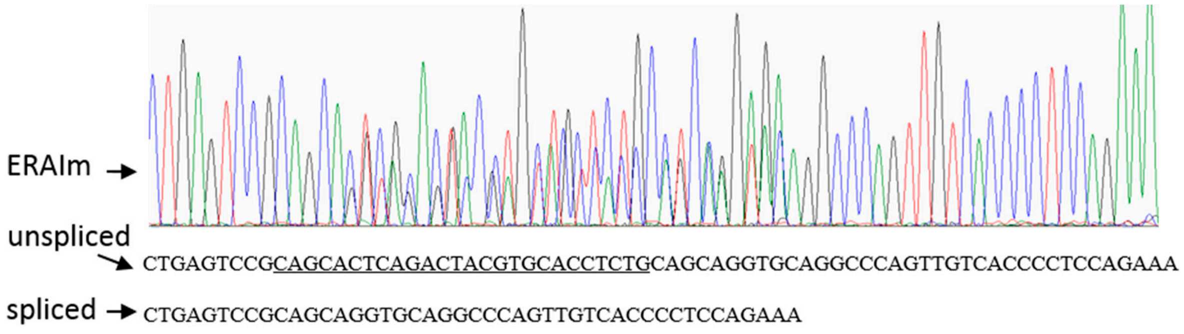

Figure S2. The sequence of unspliced ERAIm and spliced ERAIm. (The black line representative the cut off sequence).

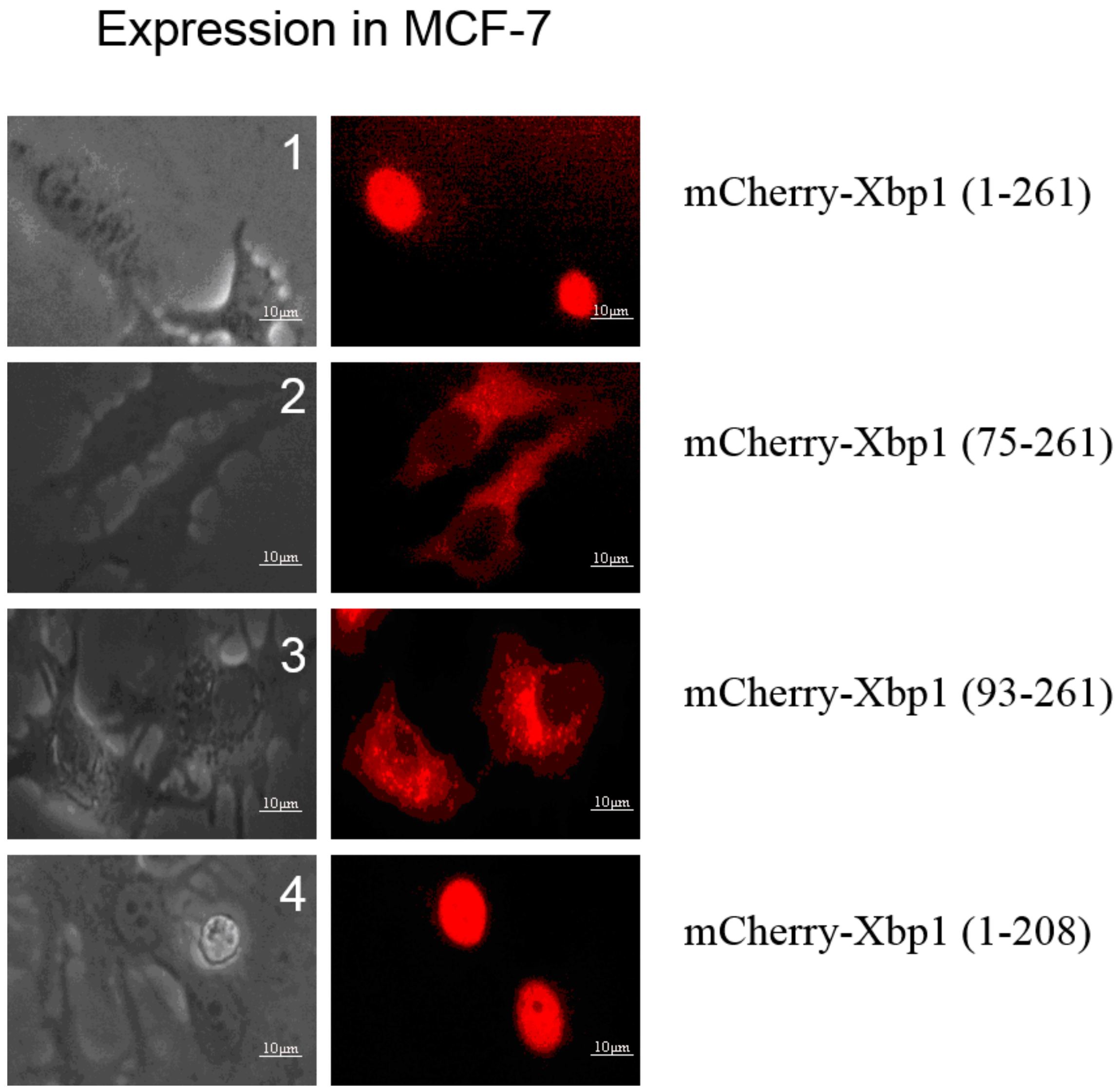

Figure S3. Expression of these mCherry-tagged XBP1 fragments in MCF-7 cells. (**1**) Expression of mCherry-Xbp1(1–261) in MCF-7; (**2**) Expression of mCherry-Xbp1 (75–261) in MCF-7; (**3**) Expression of mCherry-Xbp1(93–261) in MCF-7; and (**4**) Expression of mCherry-Xbp1(1–208) in MCF-7
